# Antimicrobial resistance and mechanisms of epigenetic regulation

**DOI:** 10.3389/fcimb.2023.1199646

**Published:** 2023-06-14

**Authors:** Xinrui Wang, Donghong Yu, Lu Chen

**Affiliations:** ^1^ Medical Research Center, Fujian Maternity and Child Health Hospital, College of Clinical Medicine for Obstetrics and Gynecology and Pediatrics, Fujian Medical University, Fuzhou, Fujian, China; ^2^ National Health Commission Key Laboratory of Technical Evaluation of Fertility Regulation for Non-Human Primate, Fujian Maternity and Child Health Hospital, Fuzhou, Fujian, China

**Keywords:** antimicrobial resistance, epigenetics, DNA modification, rRNA methylation, non-coding RNAs, epigenetic drugs

## Abstract

The rampant use of antibiotics in animal husbandry, farming and clinical disease treatment has led to a significant issue with pathogen resistance worldwide over the past decades. The classical mechanisms of resistance typically investigate antimicrobial resistance resulting from natural resistance, mutation, gene transfer and other processes. However, the emergence and development of bacterial resistance cannot be fully explained from a genetic and biochemical standpoint. Evolution necessitates phenotypic variation, selection, and inheritance. There are indications that epigenetic modifications also play a role in antimicrobial resistance. This review will specifically focus on the effects of DNA modification, histone modification, rRNA methylation and the regulation of non-coding RNAs expression on antimicrobial resistance. In particular, we highlight critical work that how DNA methyltransferases and non-coding RNAs act as transcriptional regulators that allow bacteria to rapidly adapt to environmental changes and control their gene expressions to resist antibiotic stress. Additionally, it will delve into how Nucleolar-associated proteins in bacteria perform histone functions akin to eukaryotes. Epigenetics, a non-classical regulatory mechanism of bacterial resistance, may offer new avenues for antibiotic target selection and the development of novel antibiotics.

## 1 Introduction

The discovery and widespread use of antibiotics have greatly advanced modern medicine, significantly improving the treatment of bacterial infections. However, long-term exposure to antibiotics can pose a serious risk of antimicrobial resistance (AMR), where pathogenic microorganisms become resistant to the drugs. The emergence of AMR is a growing concern, particularly with the increasing detection of clinical resistant bacteria. According to the 2019 U.S. Antibiotic Resistance Threat Report, antibiotic-resistant bacteria and fungi are responsible for over 2.8 million infections and 35,000 deaths annually in the USA alone (Centers for Disease Control and Prevention, 2019). Furthermore, predictive statistical models from the Institute for Health Metrics and Evaluation at the University of Washington, estimate that there may have been 4.95 million deathes worldwide in 2019 due to AMR (Antimicrobial Resistance Collaborators, 2022). Clearly, AMR has become a critical threat to global public health security, compounded by the onset of the post-antibiotic age and the inappropriate use of antibiotics.

**Figure 1:**
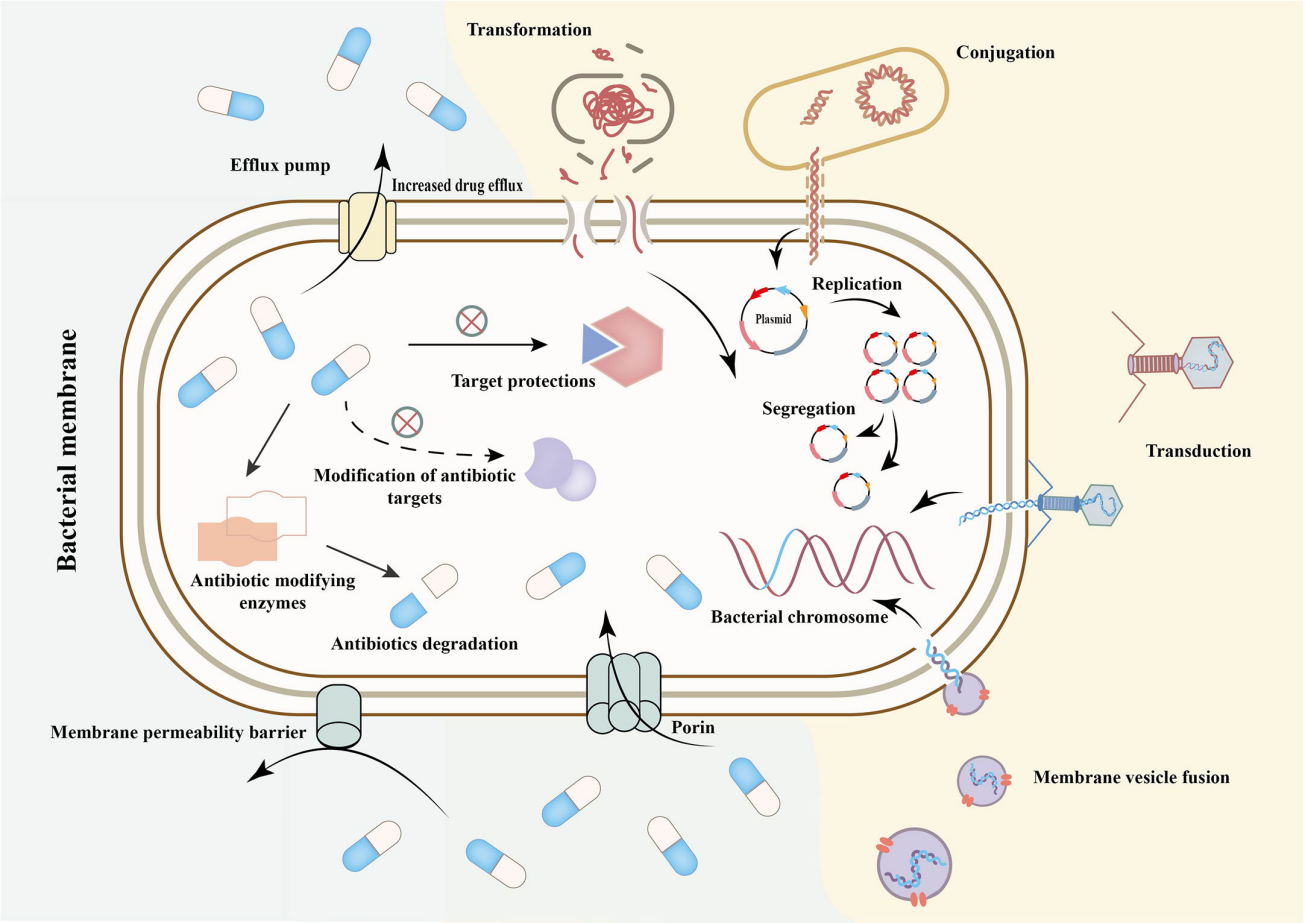
Mechanism of antimicrobial resistance and its transmission. Transformation is the intra- and inter- species exchange of naked DNA released by cell lysis or gene sequences actively effluxed by some bacteria. Conjuction is the direct transfer of DNA molecules (such as plasmids) from donor bacteria to recipient bacteria through the pipeline formed by sex pilus. Transduction is the transfer of DNA from donor bacteria to recipient bacteria by bacteriophages (Depardieu et al., 2007; Zhang, 2014; Yuan et al., 2022). Membrane vesicle fusion means that vesicles secreted which includes nucleic acids, enzymes and drug resistance genes and other substances, can enter another bacteria or host cells through direct fusion with host cell membrane or endocytosis (Depardieu et al., 2007; Zhang, 2014; Yuan et al., 2022). The outer membrane porin mediates the entry and exit of antibiotics into and out of bacteria as a permeability barrier. When the porin is missing or reduced, some antibiotics reduce influx and the host bacteria become resistant. The production of antibiotic hydrolases, inactivating enzymes and modifying enzymes can lead to the inactivation of antibiotics. The mutation or modification of related targets makes it impossible for antibiotics to bind to the corresponding sites to play a bactericidal or bacteriostatic role (Depardieu et al., 2007; Zhang, 2014; Yuan et al., 2022).

Despite more than 80 years of antibiotics use, bacteria have evolved AMR mechanisms over billions of years that allow them to escape the impact of antibiotics (Hall and Barlow, 2004). The classical AMR mechanisms include chromosomal resistance, changes in cell membrane permeability, enzyme production, target modification or mutation, active efflux pump system changes, and horizontal or vertical transfer of AMR genes (**Figure 1**) (Cox and Wright, 2013; Yelin and Kishony, 2018). These mechanisms primarily involve well-documented biochemical mechanisms and gene alterations, which are diverse, specific and heritable. However, in addition to genome changes, environmental factors and genetic context also impact the development of AMR. Antibiotics can have multiple activities, including as a resistant inducer, an inducer of resistance determinant dissemination, and an antibacterial agent (Depardieu et al., 2007). Studies demonstrate that antibiotics can induce epigenetic changes in bacterial resistance, indicating the role of epigenetics (Motta et al., 2015). While much research has focused on classical AMR mechanisms, these mechanisms fall short in explaining the emergence and spread of drug resistance due to factors such as bacterial adaptive evolution, heterogeneity, and late retention (Depardieu et al., 2007; Depardieu et al., 2007; Zhang, 2014; Becker et al., 2018; Nolivos et al., 2019; Lv et al., 2021; Yuan et al., 2022). Therefore, epigenetics may provide useful answers to these questions.

There has been growing interest in non-classical models of epigenetic-mediated bacterial AMR in recent years. In this review, we will explore the latest research on AMR in the field of epigenetics, with a focus on how epigenetic regulation influences the emergence of AMR, as well as how epigenetic regulators can reverse epigenetic phenomena and eliminate AMR. This is critical for understanding the mechanisms of AMR and for developing the potential of epigenetic regulators as direct or indirect targets for new drug therapies.

## 2 What is epigenetics?

Epigenetics refers to the study of the heritable phenotypic changes in an organism that are caused by environmental factors and genetic context, without any alterations to the DNA sequence. Epigenetic research is broadly divided into two categories (Willbanks et al., 2016): (1) Regulation of selective gene transcription, which includes DNA methylation, histone modification, chromatin remodeling and DNA phosphorothioation; (2) Post-transcriptional gene regulation, which includes regulation by non-coding RNAs (ncRNAs), RNA modification, and nucleosome positioning.

Prokaryotes have a circular, double-stranded DNA chromosome without histones, which distinguishes them from eukaryotes and ancient karyotes. This lack of key elements, such as histones and nucleosomes, that can modify DNA structure makes the epigenetic regulation mode of prokaryotes relatively simple.

### 2.1 DNA modification

#### 2.1.1 DNA methylation

**Figure 2:**
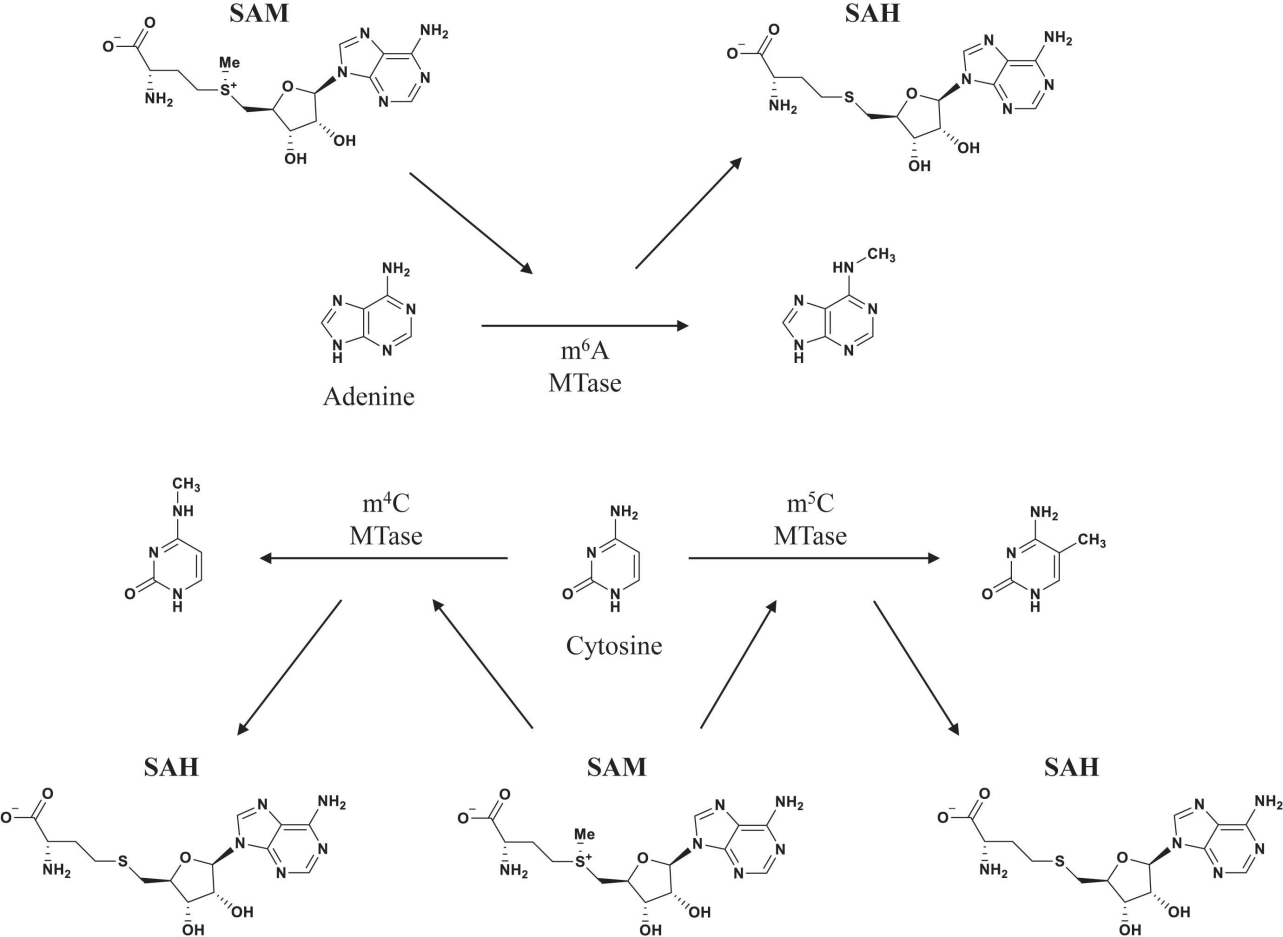
Position of DNA methylation. Adenine can add methyl at N6. Cytosine can add methyl at either endocyclic (C5) or exocyclic (N4) (Kumar et al., 2018).

In contrast to eukaryotes, bacteria lack a complete nucleus, which initially led to the theory that DNA methylation was the only type of bacterial epigenetic mechanism (Ghosh et al., 2020). Bacterial DNA methylation has been extensively studied over the past half century, revealing its involvement in chromosome replication, DNA degradation, mismatch repair, gene expression regulation, and other important physiological activities (**Table 1**) (Heusipp et al., 2007; Muhammad et al., 2022). Bacteria have three major forms of DNA methylation: 5-methylcytosine (m^5^C), N6-methyladenosine (m^6^A), and N4-methylcytosine (m^4^C). DNA methyltransferase (MTase) add methyl groups to specific DNA locations, such as the C5 or N4 position of cytosine and the N6 position of adenine (**Figure 2**) (Dunn and Smith, 1955; Holliday and Pugh, 1975). The most commonly known DNA MTases are associated with the restriction-modification (R-M) system, which is a widely known defense mechanism in bacteria. While m^5^C and m^6^A are found in most bacteria, m^4^C is specific to bacteria and archaea (Sánchez and Casadesús, 2020).

**Table 1:** Summary of bacterial epigenetics through DNA and RNA modifications.

Modifications	Type	Enzymatic Systems	Functions	Examples
DNA	Methylation	R-M system	Defense mechanism, regulate gene expression, virulence, biofilm formation	M.EcoGII, ModS, ModM, ModA, M.HpyIII, M2.HpyAII
		Orphan methyltransferases	Maintain *Eco*RII plasmid stability, DNA repair, chromosome replication, Adenine and Cytosine methyltransferases cause regulation of cell cycle	Dam, CcrM, Dcm, VchM, YhdJ,
	Phosphorothioation	DNA degradation	Defense mechanism, oxidative stress, balance intracellular redox homeostasis, influence the transcriptional efficiency	*dndABCDEFGH*
RNA	Methylation	N^6^-methyladenosine modification, N^1^-methyladenosine modification, 2-methylthiocytidine modification, 5-methylcytosine modification	Regulate RNA stability, localization, transport, splicing, antibiotic resistance and translation	RlmF, RlmJ, RlmCD
	Non-coding RNAs	Suppress or activate translation	Prevent RNA degradation	Fino/ProQ family, CsrA/RsmA family, OmpACF, MicACF

#### 2.1.2 DNA phosphorothioation

**Figure 3:**
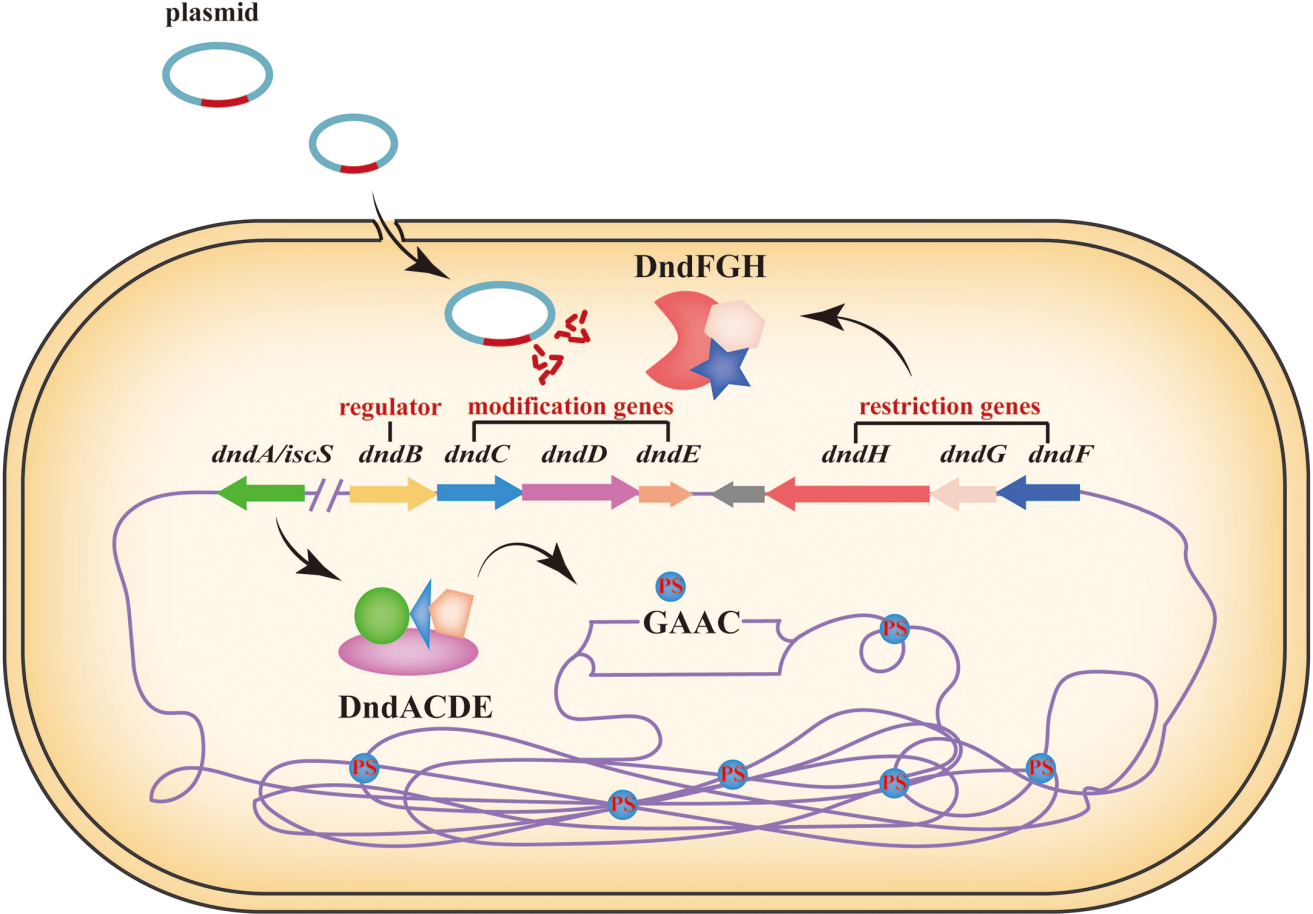
DNA phosphorothioation modification simple diagram. Based on R-M system, DNA PT modification recognize and restrict non-PT-protected foreign DNA, such as plasmids. The sulfur is transferred from L-cycsteine to DndA, and then to the cysteine residues in DndC and through DndDE complex protein to insert into the DNA backbone (Tang et al., 2022). DndB function as a negative regulator controlling the expression of *dndCDE*. DndFGH function as a restriction module to affect the acquisition of exogenous DNA (Wang et al., 2019).

In addition to DNA methylation, DNA modifications also include DNA phosphorothioation (PT) modification, which is a lesser known defence system that works in a way similar to that of the R-M system (**Table 1**) (Wang et al., 2019; Muhammad et al., 2022). PT modification, in which the nonbridging oxygen in the phosphate moiety of the DNA sugar-phosphate backbone is replaced by sulfur, was originally developed *via* chemically synthesized for decades (Tong et al., 2018). However, some research have discovered that PT modification can occur naturally in bacteria (Zou et al., 2018). Previously, it has been reported that DNA PT system consists of two parts: a five-gene *dndABCDE* cluster function as the M component to control DNA modification in a stereo- and sequence-selective manner, whereas products of the *dndFGH* cluster function as the R component to distinguish and restrict non-PT-protected foreign DNA (Tong et al., 2018). Among them, *dndA* possesses cysteine desulfurase activity and assembles DndC in bacteria (An et al., 2012). The IscS (a DndA homolog) can perform the same function as DndA to collaborate with DndBCDE in generating DNA PT modification (An et al., 2012). DndB can bind to the promoter region of the *dnd* operon to regulate the transcription of *dnd* genes. *dndCDE* function as modification genes: DndC is an iron-sulfur cluster protein that has ATP pyrophosphatase activity; DndD has ATPase activity and possibly provide energy for PT modification, and DndE is involved in binding nicked dsDNA (Hu et al., 2012; Wang et al., 2019). According to some research, the defence mechanism of PT modification has been revealed roughly (**Figure 3**). At first, the DndB function as a regulator to make response of environmental or cellular cues, and binds to the promoter region of the *dnd* operon. The DndA/IscS, DndC, DndD and DndE form a protein complex. Under the action of DndA/IscS, L-cysteine is used as a substrate to generate a persulphide group. Then, the sulphur is transferred to the DndACDE complex to complete the DNA PT modification (Wang et al., 2019). DNA PT modification has been reported in many bacteria. Except for function the similar way as the R-M system, DNA PT modification also plays important roles in antioxidant defenses, cellular redox homeostasis maintenance, environmental stress resistance, antibiotic resistance and cross talk with DNA methylation modification (Xie et al., 2012; Gan et al., 2014; Wu et al., 2017; Wu et al., 2020; Xu et al., 2023).

### 2.2 Histone modification

Histone modification is a significant epigenetic modus that plays an important role in regulating gene expression. German scientist Kossel discovered histones in the nucleus in 1884, but it wasn’t until the 1960s that their biological significance began to be investigated in depth (Doenecke and Karlson, 1984; Verdin and Ott, 2015). Histones are structural proteins that make up eukaryotic nucleosomes, which are essential for maintaining chromosomal structure and negative regulation of gene expression (Muhammad et al., 2020). Histone modification can involve methylation, acetylation, phosphorylation, and ubiquitination, each of which performs different functions (Zhang et al., 2020). Notably, bacterial genomes are packed into nucleoids through nucleoid-associated proteins (NAPs) in distinct cytoplasmic regions, rather than having a membrane-bound nucleus like eukaryotic cells (Muhammad et al., 2022).

Mounting evidence supports the idea that NAPs play crucial roles in DNA structuring and can perform functions similar to eukaryotic histones (Swinger and Rice, 2007; Stojkova et al., 2019; Amemiya et al., 2021). These structural proteins have important regulatory functions, including in bacterial virulence and pathogenesis (**Table 2**). NAPs form numerous aggregated structures with bacterial genomic DNA and participate in processes such as replication, separation, translation, and repair of prokaryotic genomic DNA. Among the primary NAPs studied are histone‐like protein (HU), leucine-responsive regulatory protein (Lrp), virulence factor transcriptional regulator (Mga*Spn*) and Histone-like nucleoid-structuring (H-NS) (Casadesús and Low, 2006; Xiao et al., 2021; Ziegler and Freddolino, 2021; Ramamurthy et al., 2022; Stojkova and Spidlova, 2022).

**Table 2:** Bacteria exert epigenetic regulation through nucleoid-associated proteins.

Bacterial Species	NAPs	Functions	References
*Francisella tularensis*	HU	Regulates the adaptive growth of bacteria and resistance to oxidative stress	(Stojkova et al., 2018; Pavlik and Spidlova, 2022)
*Streptococcus pneumoniae*	HU, Mga*Spn*	Maintains DNA supercoil, regulates bacterial viability and virulence	(Solano et al., 2016; Ferrándiz et al., 2018)
*Escherichia coli*	HU, Lrp, H-NS	Promotes bacterial invasiveness and replication in host cells, accelerates phagosome escape; regulates metabolism, virulence, exercise, nutrient transport, stress tolerance and antibiotic resistance.	(Koli et al., 2011; Ziegler and Freddolino, 2021; Norris et al., 2022)
*Porphyromonas gingivalis*	HU, IHF	Regulates biofilm formation	(Rocco et al., 2017)
*Salmonella*	Fis	Regulates the supercoiling response to bacterial growing in macrophages and virulence	(C et al., 2006)

### 2.3 RNA modification

RNA modification is an emerging area of research that has gained significant attention in recent years, which is conceptually analogous to the modifications of DNA and protein. Along with DNA methylation, RNA modification is widely found in both bacteria and eukaryotes, and over 100 types of RNA modifications have been identified, including m^6^A, N1-methyladenosine (m^1^A), m^5^C, and 2-methylthiocytidine (ms^2^C) (Lopez et al., 2020). These modifications have been shown to play a critical role in regulating RNA stability, localization, transport, splicing, and translation, ultimately affecting gene regulation and biological function (Shi et al., 2019). RNA modifications are distributed on various RNA molecules, including transfer RNA (tRNA), messenger RNA (mRNA), ribosomal RNA (rRNA) and other small RNA species such as ncRNAs. RNA modification is almost found in tRNA (Jackman and Alfonzo, 2013). Though, not as common as in tRNA, rRNA contain numerous distinct types of post-transcriptional modifications, especially rRNA methylation. Research has shown that rRNA methylation can impact antibiotic resistance development, as many antibiotic targets are located on the ribosome and ncRNAs frequently adopt central roles in regulatory networks (Laughlin et al., 2022; Wang et al., 2022; Papenfort and Melamed, 2023). Of those, RNA methylation and ncRNAs modification have been reported as the most frequent type of modification in a wide range of bacteria (**Table 1**). In this section, we will discuss the research of rRNA methylation and ncRNAs in bacterial resistance.

#### 2.3.1 Ribosomal RNA methylation

rRNA, a conserved macromolecule, is a structural component of the most abundant cellular molecule, the ribosome. In bacteria, ribosomes are composed of 16S, 23S, 5S rRNA and proteins. In eukaryotic cells, ribosomes are composed of 28S, 5S, 5.8S, 18S rRNA and proteins. In ribosomes, the rRNA is the main structural component and the core of structure and function, including (1) Synthesizing amino acids into peptide chains under the guidance of mRNA; (2) Providing binding sites for a variety of protein factors; (3) Having the activity of peptidyl transferase; (4) Providing binding sites for tRNA; (5) Targets of some antibiotics (Korobeinikova et al., 2012; Tafforeau, 2015; Srinivas et al., 2023). These functions are under tight transcriptional control to serve to meet cellular needs. Therefore, rRNA from all organisms undergoes post-transcriptional modifications that increase the diversity of its composition and activity.

Methylation of rRNA is a ubiquitous feature, and takes place during ribosomal biogenesis either by enzymes guided by an antisense small nucleolar RNA (snoRNA) or conventional protein enzymes (Lopez et al., 2020). Generally, rRNA methylation may promote the conformational rearrangement of rRNA, and regulate ribosome biogenesis and post-transcriptional modification (Wang et al., 2020). There are 25 rRNA modifications have been found in the 23S rRNA, including 13 methylations in *Escherichia coli* (*E. coli*) *(* Sergeeva et al., 2015). Wang et al. found that the absence of a single methylation in 23S rRNA affected 50S assembly and impaired translation initiation and elongation (Wang et al., 2020). In addition, rRNA methylation has emerged as a significant mechanism of AMR in pathogenic bacterial infections, such as aminoglycoside and macrolide resistance (Bhujbalrao et al., 2022; Srinivas et al., 2023).

#### 2.3.2 Non-coding RNAs

Post-transcriptional gene regulation, which includes ncRNAs, is another important epigenetic modification. There are various types of ncRNAs: including housekeeping ncRNAs such as tRNA, rRNA, and regulatory ncRNAs such as micro RNA (miRNA) and long non-coding RNA (lncRNA) (Gusic and Prokisch, 2020). These RNAs play significant roles in transcription and translation, and in eukaryotes, they are involved in regulatory processes such as development, cell death, and chromosomal silencing. Although three regulatory RNAs contained *E. coli* 6S RNA, Spot 42 and the eukaryotic 7SK RNA were first discovered by sequencing in the 1970s, but were uncharacterized until decades later (Griffin, 1971; Delihas, 2015). Until the 1980s, the *E. coli micF* RNA gene was the first regulatory RNA discovered and characterized. Recent research has shown that ncRNAs regulate various cellular processes in bacteria, including multidrug resistance, glucose metabolism, and biofilm formation (Hirakawa et al., 2003; Vanderpool and Gottesman, 2004; Zhao et al., 2022). As a result, the regulatory mode of ncRNAs has become a major focus in the bacterial regulatory network.

## 3 Bacterial epigenetics mediating antibiotic resistance

**Figure 4:**
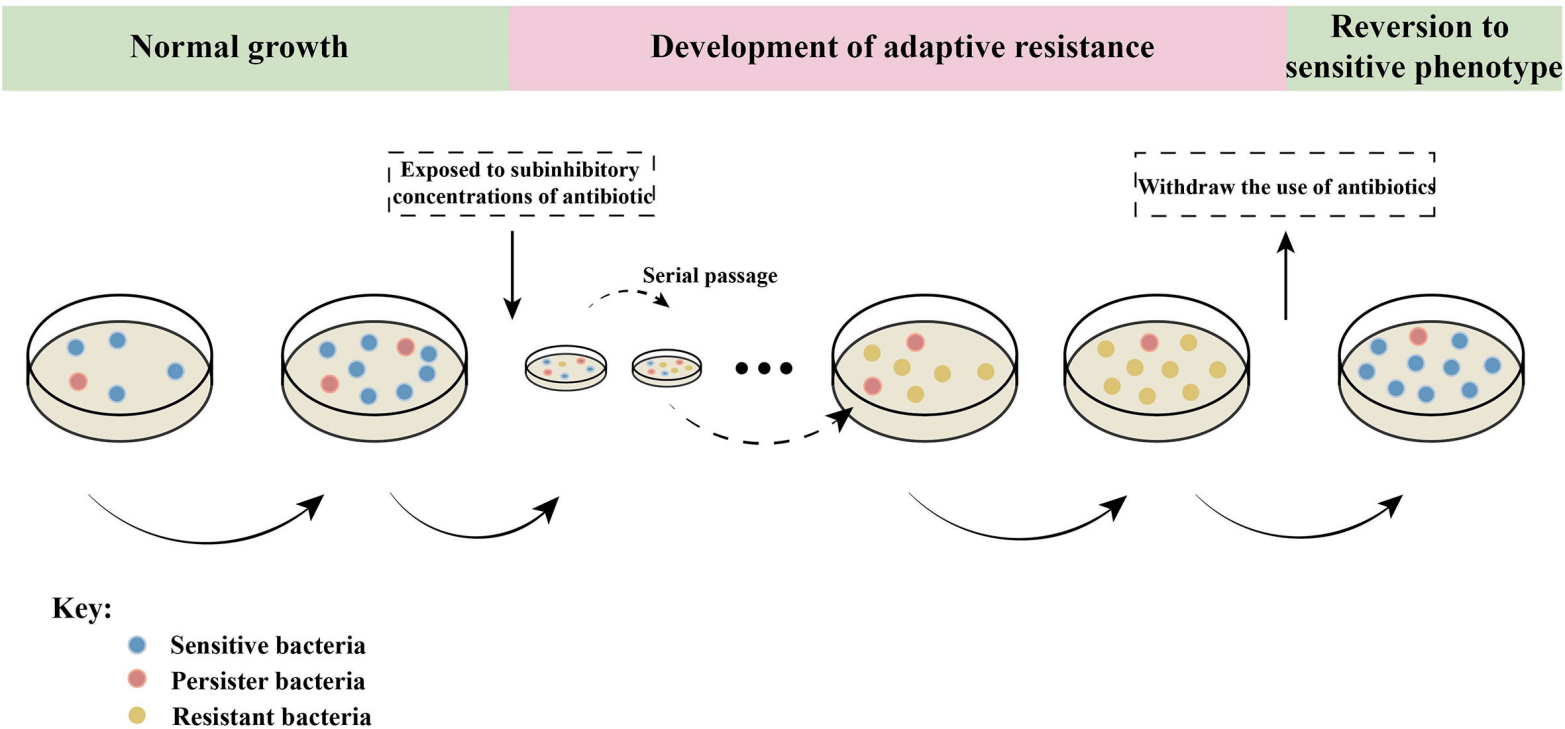
Epigenetic effects on adaptive resistance. When bacteria are continuously exposed to sub-inhibitory concentrations of antibiotics, they undergo adaptive evolution and gradually acquire resistance, which can be inherited. When antibiotics are withdrawn, the bacteria with adaptive resistance phenotype will immediately return to sensitivity (Marinus and Casadesus, 2009; Ghosh et al., 2020). Persistent bacteria are only a small part of the bacterial community that is stunted or slow to grow. Persistent bacteria can survive without mutation when exposed to antibiotic pressure (Marinus and Casadesus, 2009). These indicate that bacterial adaptive resistance is epigenetically regulated.

Bacteria have evolved to adapt to the environment over time, leading to increased antimicrobial resistance (AMR) or tolerance upon long-term exposure to antibiotics. Interestingly, bacteria can quickly restore susceptibility after returning to a normal antibiotic exposure (**Figure 4**). It is evident that gene mutations alone can not adequately explain this phenomenon.

Recent research has shown that bacteria can change the phenotypes of AMR through epigenetic intrinsic heterogeneity and transiently without the need for gene mutations (Foster, 2007; Adam et al., 2008). In order to adapt the environmental stress and ensure survival, bacteria has envolved molecular mechanisms for generating variation, such as *Helicobacter pylori* (*H. pylori*), *Haemophilus influenzae* (*H. influenzae*) and *Neisseria gonorrhoeae* (*N. gonorrhoeae*) (De et al., 2002; Srikhanta et al., 2009; Srikhanta et al., 2011). One mechanism is phase-variation, which is to randomly switch the expression of individual genes to generate a phenotypically diverse population to adapt to challenges (Seib et al., 2020). Genes can phase-vary by various of genetic mechanisms. Some studies consider that phase-variation is the high frequency reversible on/off switching of gene expression to evade antibiotic effects (Srikhanta et al., 2011). It has been reported that one way by which bacteria modulate the genes related to phase variation is *via* DNA hypermethylation or hypomethylation. However, variation in the length of hypermutable simple sequence repeats (SSRs) are a important source of phase variation, which facilitates adaptation to changing environments, immune and antibiotic escape of pathogens (Zhou et al., 2014; Pernitzsch et al., 2021). Recent studies have found that RepG (regulator of SSRs) ncRNA mediates the G-repeat length (rather than ON/OFF) and gradual control of lipopolysaccharide biosynthesis to affect AMR in *H. pylori* (Pernitzsch et al., 2021). Therefore, phenotypic variation, selection, and inheritance are necessary for evolution of bacteria. In this chapter, we summarize studies discussing the role of epigenetics in regulating AMR.

### 3.1 DNA modification

#### 3.1.1 DNA methylation

Bacterial DNA methylation plays a vital role in epigenetic regulation by controlling gene expression, genome modification, virulence, mismatch repair, transcriptional regulation, cell cycle control, and AMR (Marinus and Casadesus, 2009). The most well-known DNA MTases are associated with the defense mechanisms in bacteria known as restriction-modification systems (R-M systems). R-M systems prevent lethal cleavage of intracellular DNA by identifying their own DNA and methylating the same sequence as the restriction endonuclease cleavage site (Ghosh et al., 2020). However, foreign DNA such as plasmids carrying AMR genes, transposons, and insertable sequences cannot be methylated and will be recognized and degraded by endonucleases of the R-M systems. This defense mechanism can be circumvented if the foreign DNA carries a homolog methylase with the same specificity, and the sequence will be inserted into the genomic locus rather than degraded (Casadesús and Low, 2006; Ishikawa et al., 2010). This mechanism could explain why plasmids, phages, transposons, integrons, and gene islands can insert into bacterial genomes and contribute to the widespread dissemination of AMR genes.

**Figure 5:**
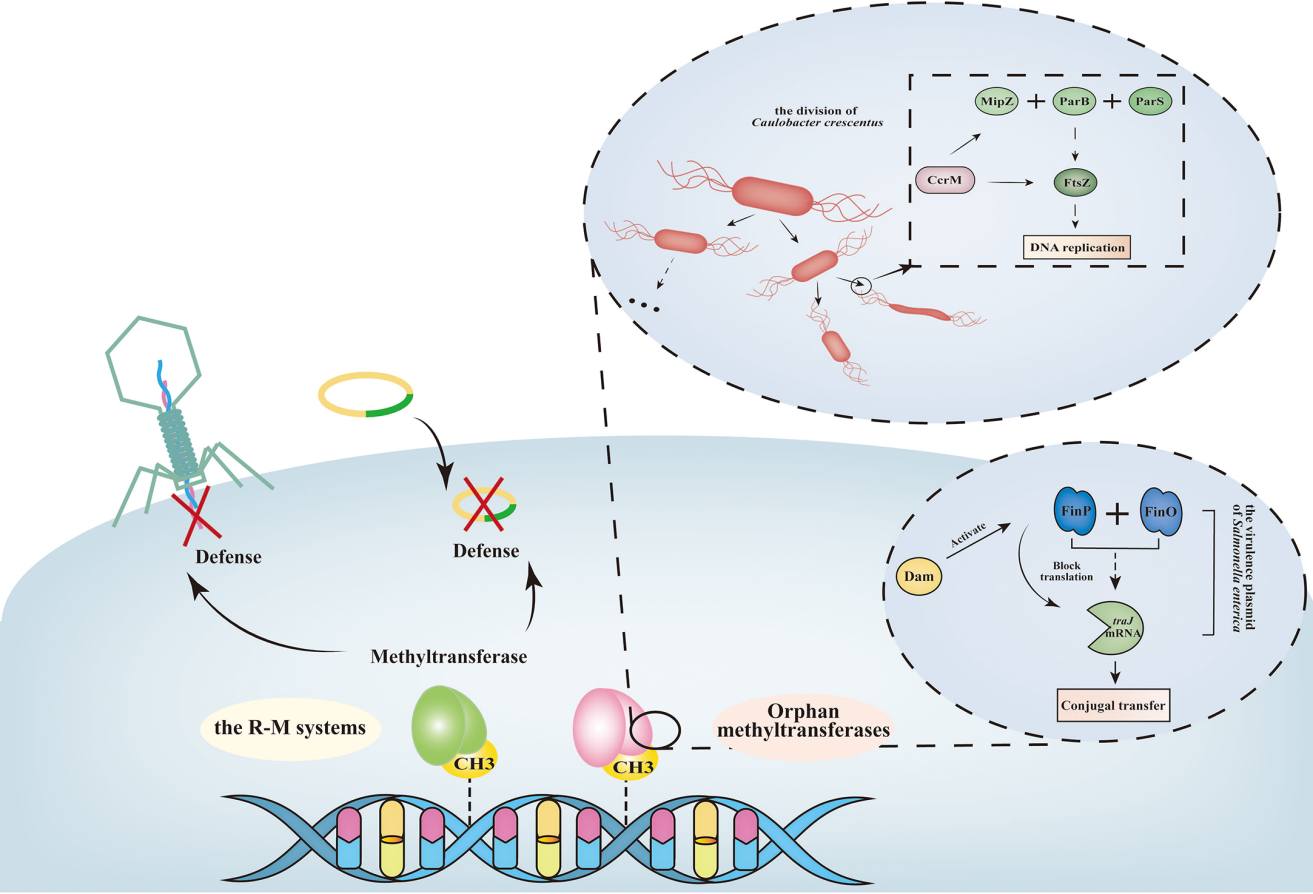
Overview of the function of bacterial DNA methylation. The R-M systems function as a barrier to recognize host genome and defenses foreign DNA, such as phage, plasmid (Phillips et al., 2019). Unlike R-M systems, orphan methyltransferases exist with no association with any restriction enzymes, and always function as regulators of DNA replication, gene transfer. Particularly, some orphan methyltransferases are not essential for most bacteria (Srikhanta et al., 2009). FinOP system regulates the conjugal transfer operon (*tra*) of plasmids. Specifically, *traJ* activates the transcription of *tra* operon (encodes the elements of pilus and products required for mating and DNA transfer). Synthesis of TraJ is controlled by FinP, a regulator that blocks *traJ* mRNA translation, and by FinO, a regulator that maintains the stability of FinP RNA-*traJ* mRNA complex (Jen et al., 2014). Dam methylation function as a conjugation repressor by activating FinP RNA synthesis. During the cell division process in bacteria, the essential FtsZ protein polymerizes into a Z-ring like structure at the future division site (Brockman et al., 2018). MipZ protein, which co-ordinates the initiation of chromosome replication with cell division, is important for the assembly of the Z-ring. MipZ interacts with the partitioning protein ParB, which then binds to the ParS locus near the chromosomal origin (davies, 2003). CcrM methylation activates the transcriptions of *ftsZ* and *mipZ*. When lacking the CcrM enzyme, the syntheses of FtsZ protein and MipZ protein are strongly downregulated, leading to a severe defect in cell division. In *Caulobacter crescentus* Δ*ccrM* strain, most Δ*ccrM* cells are filamentous with high cell length variability and frequent membrane defects (davies, 2003).

The R-M systems are classified into four types (I, II, III and IV) based on their functional localization of restriction endonuclease (Rease), activity of MTases, and requirement for specific subunits or cofactors (Roberts et al., 2003). The R-M systems have reported to function as a barrier to horizontal gene transfer in many bacteria (**Figure 5**) (Vasu and Nagaraja, 2013; Kumar et al., 2018). Li et al. found a carbapenem-resistant hypervirulent *Klebsiella pneumoniae* (*K. pneumoniae*) strain with a *bla*_kpc_ harboured conjugative plasmid and a pLVPK-like plasmid from the patient, and the type I R-M system on plasmids protected the plasmids from cleavage (Li et al., 2020). Bubendorfer et al. concluded that R-M systems inhibited genomic integration of exogenous sequencs, while they pose no effects to homeologous recombination in *H. pylori* (Bubendorfer et al., 2016).

The type I and III includes genes encoding the DNA MTase *mod*. Many studies have described that *mod* gene-mediated DNA methylation can regulate phase-variable expression associated with various resistant clinical strains (**Table 3**) (Phillips et al., 2019). For instance, the ability of *N. gonorrhoeae* to form biofilms is affected by allele *modA13* ON/OFF switching (Srikhanta et al., 2009); *Neisseria meningitidis* susceptibility to ceftazidime and ciprofloxacin result from ON/OFF of *modA11* and *modA12* OFF switching (Jen et al., 2014). A typical *H. influenzae* expressing *modA2* MTase produces more biofilms in an alkaline environment than *modA2*-deficient populations, and these biofilms have a larger biomass and less apparent structure (Brockman et al., 2018). Bacterial biofilms and AMR are closely connected. Biofilms are organized multicellular communities surrounded by an extracellular polymeric substances and can decrease bacterial metabolism, growth rate, and resistance to antibiotic penetration, all of which contribute to biofilm resistance (davies, 2003). Even in *Streptococcus suis*, Tram et al. found biaphasic switching of phase-variable DNA MTase ModS2 results in the expression of distinct phase varions. Proteins involved in general metabolism increased expression in ModS2 ON. Adversely, a glyoxalase/bleomycin resistance/extradiol dioxygenase family protein which has been described as involved in resistance to beta-lactam and glycopeptide antibiotics was upregulated in strains that did not express ModS2 OFF (Tram et al., 2021).

**Table 3:** Phase-variation of gene expression through DNA methyltransferases.

Bacterial Species	Type	Name	Number of alleles	Functions	References
*Haemophilus influenzae*	Type III R-M systems	*modA*	21	Antibiotic resistance, biofilm formation, immunoevasion and virulence	(Atack et al., 2015)
*Neisseria* species	Type III R-M systems	*modA*, *modB*, *modD*	8, 19, 10	Resistance to oxidative stress, biofilm formation, antibiotic resistance and survival	(Tan et al., 2016)
*Helicobacter pylori*	Type III R-M systems	*modH*	21	Colonization, persistent infection, motility	(Srikhanta et al., 2011)
	Type HpyAII R-M system	*M2.hpyAII*	–	DNA uptake, lipopolysaccharide profile, membrane Components, virulence, evolutionary fitness and adhesion	(Kumar et al., 2018)
*Moraxella catarrhalis*	Type III R-M systems	*modM*	6	biofilm formation, fitness cost of resistance, survival, colonization, infection, and protection against host defenses	(Blakeway et al., 2019)
*Streptococcus suis*	Type III R-M systems	*modS*	3	ABC transporters, alkylphosphonate utilization, transcriptional repressor, resistance to antimicrobials	(Tram et al., 2021)
	Type I R-M systems	*hsdS*,	4	Adhesion, virulence	(Atack et al., 2018; Roodsant et al., 2023)
*Streptococcus pneumoniae*	Type I R-M systems	*hsdS*	3	Adhesion, invasive infection, plasmid transformation rates and colony morphology	(Debroy et al., 2021)
*Escherichia coli*	Orphan methyltransferases	*dam*	–	Alter the *pap* promoter to influence the affinity of the *lrp* regulatory protein for DNA,	(Hernday et al., 2002; Zamora et al., 2020)

In addition to the well-known R-M systems, there exists a group of bacterial DNA MTases called orphan MTases, which function independently without association with any R-M system (Ishikawa et al., 2010). Orphan MTases are unique, as they do not have functional counterparts in the restriction enzyme (Reases) family. The common categories of orphan MTases include DNA adenine methyltransferase (Dam), cell cycle regulated methyltransferase (CcrM) and DNA cytosine methyltransferase (Dcm). Bacteria exhibit complex stress responses when exposed to antibiotics, leading to the phenomenon of adaptive resistance. Recent research has revealed that these three orphans MTases play a crucial role in regulating adaptive resistance and the genetic pathways involved in drug sensitivity.

##### 3.1.1.1 DNA adenine methyltransferase

Dam was the first orphan MTase identified in *E. coli*, where it modifies 5′-GATC-3′ sites (Marinus and Morris, 1973). Studies have shown that Dam-mediated DNA methylation is crucial for bacterial survival under antibiotic stress, and *E. coli* K12 Δ*dam* strains exhibit increased sensitivity to beta-lactams and quinolones (Cohen et al., 2016). Epigenetic factors, such as Dam methylation or the regulation of efflux pump expression, have been suggested to contribute to adaptive AMR (Mazzariol et al., 2000; Casadesús and Low, 2006; Adam et al., 2008). Adam et al. treated *E. coli* XL1-Blue strains with nalidixic acid and found that the expression of *dam* increased bacterial survival by approximately five-fold. This increased resistance was consistent with a two-fold rise in the expression of efflux pumps (Adam et al., 2008). Recent research has confirmed that the non-essential dam gene can be a potential target for enhancing antibiotic resistance. Chen et al. demonstrated that the *dam* deletion strain of *E.coli* MG1655 exhibited lower effective concentrations (EC50) than the wild-type strain when exposed to 20 antibiotics in five categories (Chen and Wang, 2021). This confirms that Dam plays a vital role in regulating drug sensitivity and can be utilized as a target for enhancing AMR. Dam in *Salmonella enteritidis* (*S. enteritidis*) has been found to repress the transcription of *traJ*, which encodes a transcriptional activator of the transfer (*tra*) operon of the pLST (Camacho and Casadesús, 2002). In addition, Dam activates the transcription of *finP*, which encodes a ncRNA that contributes to repression of *traJ* expression (Gorrell and Kwok, 2017). Evidence exists to suggest that in a strain with chromosomal mechanisms of quinolone resistance, a synergistic sensitization effect can be observed when the Dam methylation system and the *recA* gene were suppressed (Diaz et al., 2023).

##### 3.1.1.2 Cell cycle regulated methyltransferase

CcrM is a significant orphan MTase that modifies 5′-GANTC-3′ sites, first discovered in *Caulobacter crescentus* (*C. crescentus*). Unlike the ubiquitous Dam enzyme, CcrM expression is limited to the last stage of chromosome replication (Albu et al., 2012). In *C. crescentus*, at least four genes are directly affected by the methylation status of GANTC, including *ftsZ*, which is necessary for cell division, *ctrA* and *dnaA*, the primary regulators of the cell cycle (Reisenauer and Shapiro, 2002; Collier et al., 2007). FtsZ is an essential regulatory protein for cell division and proliferation, forming a z-ring structure at the division site. In *C. crescentus* Δ*ccrM* strain, *ftsZ* expression is significantly downregulated, leading to a severe defect in cell division (Gonzalez and Collier, 2013). The vertical transmission of heritable transfer elements carrying AMR genes is dependent on cell division and proliferation. When CcrM regulates the expression of the cytoskeleton *ftsZ* gene, it can affect bacterial division and proliferation and impact the vertical transfer of AMR genes.

##### 3.1.1.3 DNA cytosine methyltransferase

Dcm is a typical DNA MTase in *E. coli* and has two targets: 5′-CCAGG-3′ and 5′-CCTGG-3′ sites. As a result, Dcm can protect the DNA sequences from restriction enzyme ECORII activity even if the R-M system is disturbed (Gómez and Ramírez, 1993). In bacteria, Dcm is typically associated with the transcription of active genes. However, the methylation of promoter DNA is frequently associated with gene silencing in higher eukaryotes (Zemach et al., 2010). The role of Dcm in prokaryotes remains unclear, but Kahramanoglou et al. suggested that Dcm controls gene expression in the stationary phase in *E. coli (* Kahramanoglou et al., 2012). Militello et al. demonstrated that the AMR transporter SugE was overexpressed in an *E. coli* Δ*dcm* strain, indicating that Dcm may affect the drug tolerance of SugE-mediated medicines by altering the level of *sugE* gene expression (Militello et al., 2014). Furthermore, Dcm promotes plasmid loss and protects against post-segregational killing by EcoRII (which cleaves DNA at the same site as Dcm methylates) (Takahashi et al., 2002; Ohno et al., 2008).

#### 3.1.2 DNA phosphorothioation

The DNA PT modification, a novel R-M system, has been discovered widely in bacteria and archaea. As a defense barriers, DNA PT modification plays a vital part in bacterial AMR. Nonetheless, the potential role of the DNA PT modification in AMR is still unclear. By analyzing the functions of DNA PT modification in AMR with a serious of clinical pathogenic bacteria, Xu et al. demonstrated DNA PT modification reduced the distribution of horizontal gene transfer (HGT)-derived AMR genes in the genome, meanwhile the modification could suppress HGT frequence (Xu et al., 2023). To understand the mechanism of antibiotic resistance genes (ARGs) in drinking water supply systems, Khan et al. found the relative abundance of *dndB* and ARGs increased in the effluent, as well as, considered that DNA PT modification protected *mcr-1* and *bla*_NDM-1_ carrying bacteria from chloramine disinfection during the water treatment process (Khan et al., 2021). DNA PT modification can recognize and cleave unmodified exogenous DNA, such as HGT, ARGs and phage. Therefore, the modification is significant for bacteria to resist foreign invasion and maintain own genetic stability. Up to now, there is few systematic studies on AMR base on DNA PT modification, while we need to study the impact on AMR further.

### 3.2 Nucleoid-associated protein modifications

NAPs can perform histone-like functions in bacteria and affect DNA structure and transcription, unlike histones in eukaryotes. Gram-negative and Gram-positive bacteria have different NAPs, but most research focuses on Gram-negative bacteria. NAPs are essential global regulators that play a significant role in AMR (**Table 4**), as demonstrated in *Salmonella*. Yan’s research suggests that the Fis protein, known as a global regulator in *S. Typhi*, can mediated persistence by controlling glutamate metabolism (Yan et al., 2021). Additionally, the H-NS DNA binding protein can act as a transcriptional inhibitor to silence genes expression, control plasmid conjugative transfer, silence foreign genes, and inhibit conjugative transfer to reduce fitness costs (Dorman, 2007; Dorman, 2014). Cai et al. found that the IncX1 plasmid, which carries the tigecycline resistance gene *tet* (X4) and encodes the H-NS protein, results in little to no fitness cost in *E. coli* and *K. pneumoniae*. It’s also noteworthy that some plasmids can help host bacteria form biofilms and enhance virulence (Cai et al., 2021).

**Table 4:** Summary of representative Nucleoid-associated proteins in AMR.

Species	Nucleoid-associated proteins	Genes been regulated	Functions	References
*Salmonella typhi*	Fis	*gltK*, *gltJ*, *gltL*, *gltS*, *gltH* and *gltP*	Regulate glutamate metabolism to reduce persister formation	(Yan et al., 2021)
*Salmonella typhi*	H-NS, Hha, StpA	pathogenicity islands (SPIs), *pef*	Inhibite the expression of SPI2 to improve the fitness,	(Hurtado et al., 2019)
*Escherichia coli*	Fis	*fimS*, *fimA*, *fimB*, *acs, acnB*, *fum*	Function as a negative regulator in the *fimS* phase variation, enhanced growth ftness under acetate metabolism, regulate biofilm formation	(Jindal et al., 2022; Saldaña et al., 2022)
*Escherichia coli*	H-NS	*pilx1*-*11*, *taxB*, *taxC*, *actX*, *parB*	Facilitate horizontal plasmid transfer, affect the stability of plasmid	(Liu et al., 2020)
*Escherichia coli*	HU, IHF	*fim*, *pap*	Promote biofilm formation, Gp46 function as HU inhibitor	(Justice et al., 2012; Devaraj et al., 2015; Zhang et al., 2022)
*Shigella*	H-NS	*virB*	Silence the *virB* promoter and influence virulence plasmid trasnsfer	(Colonna et al., 1995)
*Acinetobacter baumannii*	H-NS	*aidA, abaI, kar, fadD, bla*_OXA-23_ *, bla*_OXA-51-like_ *, bla*_ADC_ *, bla*_GES-14_ *, carO, pbp1*, and *advA*	Regulate the expression of genes encoding efflux pumps and the formation of biofilm; modulate the expression of resistance-related genes	(Rodgers et al., 2021)
*Klebsiella pneumoniae*	H-NS	*tet* (X4),	Modulate the fitness cost of plasmids, promote the virulence and biofilm formation,	(Cai et al., 2021)
*Mycobacterium tuberculosis*	HU, HupB	*eis*, *arsR*, *marR*, *tetR*	Regulate the sensitivities of aminoglycosides, alter gene expression and phenotypic state in a subpopulation	(Zaunbrecher et al., 2009; Ghosh et al., 2016; Sakatos et al., 2018; Rodgers et al., 2021)
*Porphyromonas* *gingivalis*	HU	*ssP*, *fimA*	Disperse oral streptococcus biofilm and prevent *P. gingivalis* entry into oral *Streptococcus* biofilm	(Rocco et al., 2018)

Compared to DNA methylation, histone modification has greater plasticity. The H-NS protein can regulate the expression of genes encoding efflux pumps in multidrug-resistant *Acinetobacter baumannii* (*A. baumannii*) and down-regulate the expression of AMR genes for beta-lactams, aminoglycosides, quinolones, chloramphenicol, trimethoprim, and sulfonamides (Rodgers et al., 2021). Similarly, deleting *hns* lowers the expression of biofilm-related genes in *A. baumannii* (Rodgers et al., 2021). A recent study found that H-NS affects the stability of *bla*_NDM-1_-bearing IncX3 plasmid and inhibits its plasmid conjugative transfer in *E. coli (* Liu et al., 2020). These indicate the complexity and breadth of the regulatory network controled by H-NS for genes involved in AMR and persistence.

In view of the biofilms play a major role in some chronic and recurrent infections and are associated with the failure of antibiotic therapy, antibiotic therapy is the first -line treatment of bacterial infections (Devaraj et al., 2018). The DNA-binding (DNABII) protein family includes two well-known NAPs, integration host factor (IHF) and HU. These proteins bind to DNA with high affinity and bend it, thereby playing essential roles in the structure and function of the bacterial nucleoid (Browning et al., 2010). While IHF binds to specific DNA sequences, HU does not. In addition to their structural functions, IHF and HU are also crucial for biofilm formation and the integrity of community structure (Devaraj et al., 2015). In uropathogenic *E. coli*, both subunits of IHF aid in biofilm formation, while HupB (HUβ), one of the subunits of HU, is required for biofilm formation (Devaraj et al., 2015). IHF and HU could be potential therapeutic targets for biofilm therapy, as antimicrobial agents and the host immune system have difficulty attacking biofilms. A research has found that the HU protein subunit HupB, post-translationally modified by lysine acetylation and methylation, is a breakthrough in treating multidrug-resistant *Mycobacterium tuberculosis* (*M. tuberculosis*) *(* Ghosh et al., 2016). Mutating a single post-translational modification site eliminates a drug-resistant cell subset of isoniazid-resistant *M. tuberculosis* (Sakatos et al., 2018). Additionally, it has been reported that using anti-*Porphyromonas gingivalis* (*P. gingivalis*) HUβ antibodies to specifically target the oral *Streptococcus* biofilm for preventing *P. gingivalis* organisms from entering into preexisting biofilms formed by oral *Streptococcal* species (Rocco et al., 2018). Therefore, HU, for instance HupB, could be a promising therapeutic target for bacterial therapy. Recent research has reported that targeting HU, Zhang et al. used Gp46 (an HU protein inhibitor from phages) to inhibit HU of many resistant pathogens by occupying DNA binding site, and preventing chromosome segregation during cell division (Zhang et al., 2022).

### 3.3 RNA modification

#### 3.3.1 Ribosomal RNA methylation

RNA modifications, such as rRNA methylation, have emerged as important mechanisms associated with AMR. Ribosomes are a common target for antibiotics. Methylation of specific sites in rRNA can prevent antibiotics from binding to their target sites, thereby leading to antibiotic resistance. Thus AMR *via* rRNA methylation is one of the most common strategies adopted by multidrug resistant pathogens. One such example is 16S rRNA methylation, which is a major mechanism of aminoglycoside resistance in clinical pathogens (Tada et al., 2013; Liu et al., 2015). Two different methylation sites in 16S rRNA lead to different aminoglycoside-resistant phenotypes. Methylation of residue A1408 confers resistance to kanamycin and apramycin in *E. coli*, but sensitivity to gentamicin, while methylation of residue G1405 confers resistance to kanamycin and gentamicin, but sensitivity to apramycin (Liu et al., 2015). The multidrug resistance gene *cfr*, found in *Staphylococcus*, encodes an MTase that modifies the A2503 site in 23S rRNA, leading to resistance to antibiotics such as amide alcohols, lincomycins, oxazolidinones, pleuromutilin, and streptogramin A (Long et al., 2006). In *S. pneumoniae*, U747 methylation mediated by RlmCD promotes efficient G748 methylation by the MTase RlmA^II^ in 23S rRNA, affecting the susceptibility to telithromycin (Shoji et al., 2015). Another research indicated the erythromycin-resistance MTase methylates rRNA at the conserved A2058 position, and imparts resistance to macrolides, such as erythromycin (Bhujbalrao et al., 2022). Up to now, the number of rRNA MTases related to AMR mechanisms have increased, but the source of MTases and the exact mechanisms of AMR are still unclear.

#### 3.3.2 Non-coding RNAs

Advancements in high-throughput sequencing technology and bioinformatics have facilitated the discovery of various ncRNAs and their functions in bacteria. Recent studies have found that exposure to environmental stress, especially antibiotics, bacteria produce specific ncRNAs profiles, which may regulate the expression of downstream genes. When bacteria sense antibacterial stress, a large number of ncRNA regulators are upregulated, and one of their roles is to improve bacterial adaptation in a dynamic environment (Morita and Aiba, 2007). Thus, ncRNAs play an essential role in the bacterial regulatory network that controls the expression of bacterial genes through regulating proteins and target mRNAs. In comparison to regulatory proteins, ncRNAs are considered a better class of regulatory molecules for controlling gene expression (Toledo et al., 2007).

ncRNAs play an essential role in the regulation of bacterial gene expression and can affect AMR mechanisms. Although ncRNAs are a major form of post-transcriptional gene control in bacteria, some research indicate ncRNAs also influence transcription (Rodgers et al., 2023). For instance, Majdalani et al. found that RprA ncRNA reduced type IV secretion-mediated transfer of pSLT (*Salmonella* virulence plasmid) (Papenfort and Melamed, 2023). In particular, RrpA controls the transcription and translation of *ricI*, which encodes a membrane protein that interacts with and suppresses the anchor protein Trav of the type IV secretion apparatus (Majdalani et al., 2001). It is reported that antisense *vicR* (a kind of ncRNAs) is transcribed from the opposite strand of *vicR* mRNA and regulates the biofilm formation of *Streptococcus mutans via* affecting the production and function of VicR protein (Lei et al., 2018).

The incomplete complementary pairing of most ncRNAs with the target mRNA sequence can lead to two results: (1) Blocking the ribosome binding sites and suppressing translation; (2) Secondary structure melting, exposing the nucleose binding site and translation start site, leading to translation activation (Vogel and Sharma, 2005; Fröhlich and Vogel, 2009). Moreover, since the instabilized base pairing between the ncRNAs and their target mRNAs, the RNA chaperone protein Hfq, binding protein Fino/ProQ family, CsrA/RsmA family and other regulators usually facilitate imperfect base pairing between ncRNAs and mRNAs, leading to regulate the translation initiation frequency or the stability of target mRNAs (Liao and Smirnov, 2023; Wang et al., 2023; Yu and Zhao, 2023). In this chapter, we will explore some research on ncRNAs that regulate the mechanisms of AMR from two perspectives.

##### 3.3.2.1 Translation suppression

ncRNAs regulate bacterial cell wall or membrane to alter the sensitivity of antibiotics. Bacteria can control membrane permeability by regulating the expression of outer membrane proteins OmpF, OmpA, and OmpC. Studies have shown that ncRNAs such as MicF, MicA, and MicC inhibit the expression of these mRNAs by partial complementary pairing, interfering with antibiotic exposure (Chen et al., 2004; Udekwu et al., 2005). Therefore, ncRNAs represent a promising target for the development of new strategies to combat AMR in bacteria.

ncRNAs have been shown to affect AMR by targeting the efflux pumps. For instance, overexpression of SdsR has been found to decrease the mRNA and protein levels of the TolC,which encodes the outer membrane protein of many multidrug resistance efflux pumps, resulting in increased sensitivity to fluoroquinolones in *E. coli* (Kim et al., 2015; Parker and Gottesman, 2016). However, in *Shigella sonnei*, overexpression of SdsR leads to lower mRNA levels of *tolC* and increased survival rates at sub-MIC norfloxacin (Gan and Tan, 2019). *Pseudomonas aeruginosa* (*P. aeruginosa*) is a common source of hospital infections and has important adaption abilities to various environmental exposures (Jurado et al., 2021). A recent study found that overexpressing of AS1974 ncRNA restores the sensitivity of MDR clinical strains by down-regulating the expression of MexC-MexD-OprJ, a component of the multidrug efflux system (Law et al., 2019). On the other hand, overexpression of PA08051 and PA2952.1 ncRNAs leads to up-regulation of the drug efflux system mexGHI-opmD, resulting in increased resistance of aminoglycoside (Coleman et al., 2020; Coleman et al., 2021).

Bacterial biofilms, which are microcolonies formed by adhesion on solid surfaces or between bacteria, can secrete extracellular matrix to create a natural barrier. This multicellular-like lifestyle allows resistance to environmental and cell-intrinsic stresses, such as antibiotics exposure. For example, Falcone et al. found that based on RNA-seq analysis, the ErSA ncRNA of *P. aeruginosa* complementary pairs with *amrZ* mRNA to influence the expression of AmrZ, promoting biofilm development (Falcone et al., 2018). The RNA-binding protein ProQ has been shown to regulate mRNA-expression levels by interactions with 5′ and 3′ UTRs (Holmqvist et al., 2018). In an early study found that ProQ was necessary for robust biofilm formation, and this phenotype was independent of ProP (Sheidy and Zielke, 2013). Infections caused by *Staphylococcus aureus* (*S. aureus*) are often associated with adverse therapeutic outcomes due to various reasons, such as an antibiotic penetration barrier by bacterial biofilms (Singh et al., 2016). By sensing and responding to multifarious environmental exposure, bacteria carry out corresponding adaptive regulation. For instance, the *teg58* ncRNA have specific interaction with *argGH* mRNA (arginine biosynthesis genes) to repress arginine synthesis and biofilm formation in *S. aureus (* Manna et al., 2022). Raad et al. found that during stationary phase of *E. coli*, the 3’ UTR-derived FimR2 ncRNA interacted with CsrA, antagonizing its post-transcriptional functions of flagellar and fimbrial biosynthesis, and firmly strengthening the control of bacterial motility and biofilm formation (Raad et al., 2022).

ncRNAs affect AMR by regulating the functions of plasmids carrying resistance genes, including fitness and conjugation. HGT refers to the transfer of genes between unrelated species, which increases genetic diversity and accelerates bacterial evolution (Gogarten and Townsend, 2005). Conjugative plasmids are typical representatives of HGT and promote the spread of AMR among pathogens. Due to plasmid reception, intergration, replication and the expression of genes, the antibiotic-resistant plasmids produce fitness costs in host bacteria (San and Maclean, 2017). Therefore, it seems that plasmids gradually lost over time during bacterial evolution without corresponding antibiotic exposure. In contrast to this conjecture, antibiotic-resistant plasmids can stably persist in host bacteria for long periods without any antibiotics (Zhang et al., 2022). There may be some mechanisms that regulate the bacteria fitness cost. Some research have found that ProQ/FinO family proteins encoded by the IncI2 plasmid carrying *mcr-1*, balanced *mcr-1* expression and bacteria fitness by inhibiting plasmid copy number (Yang et al., 2021). As well as, the RNA-binding protein ProQ has identified three distinct domains, one is a large conserved N-terminal Fino-like domain (Gulliver et al., 2022). The FinO-like domain facilitates binding to the RNA, shares similar structural and functional characteristics with the FinO RNA chaperone in IncF plasmid (Pandey et al., 2020). FinO was named so to reflect its fertility inhibition function observed in IncF plasmid conjugation (Finnegan and Willetts, 1972). These plasmids regulate conjugation through RNA antisense mechanisms, whereby the *cis*-encoded ncRNA FinP inhibits protein synthesis of conjugative transfer regulator TraJ (Timmis et al., 1978; Van Biesen and Frost, 1994; El et al., 2021). The synthesis of TraJ is inhibited, and leads to higher conjugation of plasmids without FinO (El et al., 2021). El Mouali et al. found that the binding protein FinO encoded in virulence plasmid of *Salmonella* also regulated the replication of a cohabitating plasmid carrying antibiotic gene, which may suggest cross-regulation of plasmids in RNA level (El et al., 2021).

##### 3.3.2.2 Translation activation

ncRNAs affect AMR by activating translation. ncRNAs commonly down-regulate gene expression, however, also have the ability to activate genes by multifarious mechanisms in bacteria. Several ncRNAs act as direct translational activators by preventing the formation of translation-inhibited stem-loop structures through antisense pairing translation in the 5′mRNA region (Fröhlich and Vogel, 2009). After being activated by the main regulators LuxO/HapR of the quorum sensing system, the Qrr ncRNA (quorum regulatory RNAs) of *Vibrio* species binds to the chaperone Hfq and regulates downstream gene expressions (Hammer and Bassler, 2007). One of the pathways is the HapR-independent pathway: the Qrr ncRNA interaction with *vca0939* mRNA prevents formation of inhibitory stem-loop structures, allows access to ribosomes and promote translation (Hammer and Bassler, 2007). Moreover, after the translational activation, *vca0939* encodes GGDEF proteins and induces virulence factors and biofilm formation (Camilli and Bassler, 2006).

## 4 Epigenetic drugs as treatment of antimicrobial resistance

Epigenetic drugs are small molecules that have been designed or studied based on epigenetic mechanisms, such as selective transcription or post-transcriptional regulation of genes. Some epigenetic drugs have been found to alter gene expression by inhibiting specific enzymes. Given the current situation of AMR, epigenetic drugs have important implications for the treatment of infectious diseases caused by multidrug-resistant bacteria. For instance, low concentrations of SAM analogues, such as SGC0946, JNJ-64619178, and SGC8158 were found to inhibit the activity of *C. difficile*-specific DNA adenine MTase, selectively affecting biofilm and spore production and quickly eradicating *C. difficile* infection (Zhou et al., 2022). Moreover, UVI5008, a derivative of the natural substance psammaplin A, was found to reduce the DNA gyrase activity of methicillin-resistant *S. aureus*, and reverse AMR by damaging the bacterial cell wall (Franci et al., 2018). Similarly, epigallocatechin-3-gallate (EGCG) can damage the integrity of the cell wall and reverse the resistance of imipenem, tetracycline, and amoxicillin in *S. aureus* (Sudano et al., 2004; Zeferino et al., 2022). With the deepening of research, Serra et al. thought that EGCG directly interfered with the assembly of curli fimbriae into amyloid fibrils and reduced the synthesis of CsgD (activator of curli fimbriae and cellulose biosynthesis) by promoting the expression of RybB ncRNA, ultimately inhibited the formation of cell membranes and affected biofilm-mediated antibiotic resistance and host defense (Serra et al., 2016) As well as, EGCG was found to be a suitable natural drug targeting LuxS/AI-2 system of *H. pylori* by high-throughput screening and molecular dynamics simulation (Ashok et al., 2023). Zhang et al. found that EGCG prevented *Shigella flexneri* biofilm extracellular polysaccharide from forming through reducing the expression of *mdoH* gene (Zhang et al., 2023). These findings suggest that epigenetic drugs have the potential to be used as a treatment for patients with multidrug-resistant bacterial infections.

## 5 Conclusions

AMR is an ancient and natural phenomenon, that has evolved in bacteria over millions of years. While biochemical and genetic alterations are known to contribute to AMR, non-classical mechanisms such as epigenetics have recently gained attention. Bacterial epigenetics, which involves modifications to DNA and rRNA, ncRNAs, as well as nucleoid-associated proteins, has been shown to regulate the formation and enrichment of AMR. This regulatory mechanism controls gene expression switching, phase variation, bacterial tolerance, and persistent bacteria. The epigenetic regulatory mechanisms of bacteria are complex which may have long term implications. Although our current understanding of bacterial epigenetics is still limited, recent advances in sequencing technologies are enabling high-resolution mapping of epigenetic landscapes in prokaryotes, which is expected to shed light on the complex regulatory mechanisms of AMR. With the advent of the post-antibiotic era, the discovery of epigenetic mechanisms in multidrug-resistant pathogens also helps to search for antibiotic potentiators or provide new targets for the development of newer drugs.

## Author contributions

XW and DY researched data for the manuscript. LC provided conceptualization and was responsible for the first draft of the manuscript. XW provided conceptualization, review, comment and editing. All authors discussed the results and reviewed and commented on the manuscript. All authors contributed to the article and approved the submitted version.
